# Increased incident rates of antidepressant use during the COVID-19 pandemic: interrupted time-series analysis of a nationally representative sample

**DOI:** 10.1017/S0033291722001891

**Published:** 2023-08

**Authors:** Sophia Frangou, Yael Travis-Lumer, Arad Kodesh, Yair Goldberg, Faye New, Abraham Reichenberg, Stephen Z. Levine

**Affiliations:** 1 Department of Psychiatry, Djavad Mowafaghian Centre for Brain Health, University of British Columbia, Vancouver, Canada; 2 Department of Psychiatry, Icahn School of Medicine at Mount Sinai, New York, USA; 3 Faculty of Industrial Engineering and Management, Israel Institute of Technology, Haifa, Israel; 4 Department of Community Mental Health, University of Haifa, Haifa, Israel; 5 Meuhedet Health Services, Tel Aviv, Israel

**Keywords:** Coronavirus, disaster, epidemiology, public health

## Abstract

**Background:**

The COVID-19 pandemic has been associated with increased levels of depression and anxiety with implications for the use of antidepressant medications.

**Methods:**

The incident rate of antidepressant fills before and during the COVID-19 pandemic were compared using interrupted time-series analysis followed by comprehensive sensitivity analyses on data derived from electronic medical records from a large health management organization providing nationwide services to 14% of the Israeli population. The dataset covered the period from 1 January 2013 to 1 February 2021, with 1 March 2020 onwards defined as the period of the COVID-19 pandemic. Forecasting analysis was implemented to test the effect of the vaccine roll-out and easing of social restrictions on antidepressant use.

**Results:**

The sample consisted of 852 233 persons with a total antidepressant incident fill count of 139 535.4 (total cumulative rate per 100 000 = 16 372.91, 95% CI 16 287.19–16 459.01). We calculated the proportion of antidepressant prescription fills for the COVID-19 period, and the counterfactual proportion for the same period, assuming COVID-19 had not occurred. The difference in these proportions was significant [Cohen's *h* = 10^−3^ (0.16), 95% CI 10^−3^ ( − 0.71 *to* 1.03)]. The pandemic was associated with a significant increase in the slope of the incident rate of antidepressant fills (slope change = 0.01, 95% CI 0.00–0.03; *p* = 0.04) and a monthly increase of 2% compared to the counterfactual (the estimated rate assuming no pandemic occurred). The increased rate was more pronounced in women, and was not modified by lockdown on/off periods, socioeconomic or SARS-CoV-2 status. The rate of observed antidepressant fills was similar to that forecasted under the assumption of ongoing COVID-19 distress.

**Conclusion:**

These findings underscore the toll of the pandemic on mental health and inform mental health policy and service delivery during and after implementing COVID-19 attenuation strategies.

## Introduction

The global spread of the SARS-CoV-2 coronavirus, which causes COVID-19 disease, has created an unprecedented health crisis. As of 27 March 2022, the World Health Organization has recorded nearly 500 million confirmed COVID-19 cases and over 6 million deaths worldwide (World Health Organization, 2022). Despite their effectiveness in reducing COVID-19 cases and mortality (Girum, Lentiro, Geremew, Migora, & Shewamare, 2020; Haug et al., 2020; Islam et al., 2020), the public health attenuation strategies involving mask-wearing, social distancing, and stay-at-home orders (lockdowns) have profoundly influenced the daily lives of billions of people over the last 2 years while the emergence of new fast-spreading variants have caused a surge in infections, increased COVID-19 anxiety, and have raised the specter of renewed lockdowns. The impact of the COVID-19 pandemic has also led to financial hardship for many individuals and families, and ongoing economic uncertainty and strife in many countries. The combined effects of the COVID-19 pandemic have been described as a mass social trauma (Feuer, 2021) and a perfect storm of substantial risks (Reger, Stanley, & Joiner, 2020) to mental health.

Significant research efforts are underway worldwide to evaluate changes in the levels of psychological distress and psychiatric diagnoses associated with the COVID-19 pandemic amid ongoing fears that its adverse effect on mental health could persist long after the numbers of SARS-CoV-2 infections and COVID-19 disease subside (Horton, 2020; Travis-Lumer, Kodesh, Goldberg, Frangou, & Levine, 2021). Nationwide UK data demonstrate that since the beginning of the pandemic average ratings of overall mental well-being have declined and the proportion of individuals reporting depressive symptoms has nearly doubled compared to pre-pandemic levels, with social restrictions seemingly having a causal impact (Office of National Statistics, 2021a). Similar observations for anxiety and depression have been made by the US National Center for Health Statistics (NCHS, 2021) and multiple international studies assembled by the Wellcome Trust-funded COVID-Minds Network (2021).

The use of antidepressant medication during the pandemic provides complementary information, focused on the intersection between help-seeking behavior and service provision. Studies of antidepressant use derived from large-scale nationwide datasets are few, but those from Europe and the USA provide convincing evidence of increased antidepressant prescription pharmacy claims during the pandemic compared to the pre-pandemic period (Estrela et al., 2022; Express Scripts, 2020; Rabeea, Merchant, Khan, Kow, & Hasan, 2021), and suggest significant moderating effects for sex and age (NHS Digital, 2021).

As prior literature focused mostly on the initial part of the pandemic and the first phase of lockdowns, the present study aimed to examine longer-term and more granular data of antidepressant use associated with the COVID-19 pandemic. We focus specifically on the rates of new (i.e. incident) antidepressant prescription fills as a proxy for prescribing changes likely to have been triggered by COVID-19-related mental health problems. To achieve this, we leveraged data from the electronic health records (EHRs) of a health management organization (HMO) covering an unselected sample of 14% of the Israeli population nationwide.

The present study expands the literature in several ways. First, the study sample examines an unselected segment of the Israeli population served a single HMO; this sample has minimal non-inclusion biases because the legal framework for health service provision in Israel (Chinitz, Shalev, Galai, & Israeli, 1998) mandates that all citizens join a single HMO and that HMOs must offer comparable nationwide services, in terms of cost and range, without discrimination on demographic or medical grounds. Second, pharmacy services in Israel remained accessible throughout the pandemic thus minimizing biases associated with filling prescriptions. Third, in addition to the possible effects of sociodemographic features we also examine the impact of SARS-CoV-2 infection status (cases *v.* non-cases) on antidepressant use which is a particular strength of the study. Fourth, we aggregated data from 1 January 2013 to 1 February 2021, with 1 March 2020 defined as the onset of the pandemic in Israel; the dataset therefore covers 11 months of the pandemic, including all three lockdown waves in Israel, and comprises a long pre-pandemic observation period that allowed us to control for non-pandemic-related fluctuations in antidepressant fills. Fifth, data analyses were undertaken using interrupted time series (ITS) which is a powerful quasi-experimental design. Sixth, we include forecasting scenarios to test whether vaccination and easing of social restrictions would alter the incident rate of antidepressant fills. Israel launched its COVID-19 vaccination campaign on 19 December 2020. At least one vaccine dose was administered to about third of population by 1 February 2021, and about three-quarters of the population by November 2021 (Mathieu & Ritchie, 2021). Vaccination has been viewed as the most promising intervention for ending the pandemic, and by extension the associated psychological distress. This comprehensive evaluation of incident antidepressant use has the potential to uncover key drivers of the patterns observed and inform about post-pandemic service needs.

## Method

### Sample

Four Israeli non-profit HMOs provide healthcare services to the entire Israeli population under provisions laid out in the National Health Insurance Law (Chinitz et al., 1998). This legislation dictates that each HMO offers nationwide services without differences in financial or service provision. Legislation dictates that each Israeli citizen must choose to join a single HMO. It is illegal for an HMO to deny membership based on demographic or prior or existing medical characteristics. Accordingly, non-inclusion by an HMO (and hence, sample selection) would violate the legislation. The data used in the current study are from the HMO ‘Meuhedet Healthcare Services’ (hereafter Meuhedet) which serves 14% of the total population of Israel nationwide. Ethical approval was granted by the Meuhedet-associated Helsinki Institutional Review Board with a waiver of informed consent. The source population is the nationwide Meuhedet members aged over 15 years. The analysis comprised data from EHRs that were continuously collected between 1 January 2013 and 1 February 2021. The data collection interval covers the pre-pandemic period as well as most of the period of the COVID-19 pandemic in Israel, including all lockdown waves. Table 1 presents the sample characteristics at the beginning of the study in January 2013, when there were 707 798 individuals over the age of 15 years insured by Meuhedet. This number increased during the study to 852 233 by 1 February 2021 (end of data collection for the interrupted series model) and to 865 044 by 30 November 2021 (sample used to derive empirical data to confirm forecasting analyses).

**Table 1.** Sociodemographic sample characteristics at study onset

Total sample (*N*)	707 798
Sex	
Female, *N* (%)	360 947 (51.0)
Male, *N* (%)	346 851 (49.0)
Age brackets (years), *N* (%)	
15–24	162 454 (23.0)
25–34	145 748 (20.6)
35–44	144 645 (20.4)
45–54	101 612 (14.4)
55–64	80 747 (11.4)
65–74	44 641 (6.3)
75+	27 951 (3.9)
Socioeconomic status, *N* (%)	
High	102 959 (14.5)
Low	144 578 (20.4)
Medium	425 895 (60.2)
Undeterminable	34 366 (4.9)

### Study design

#### Interrupted time series

**Fig. 1.**
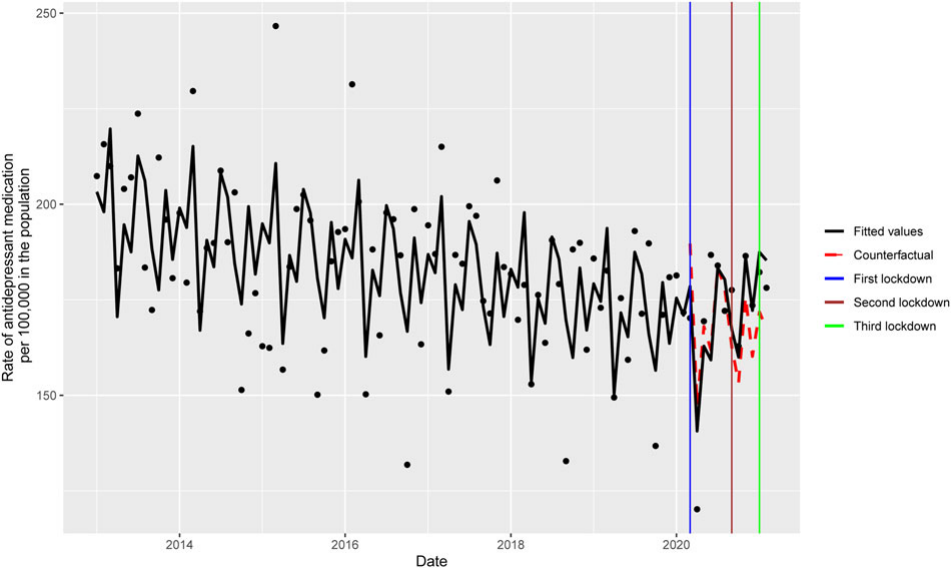
Comparison of incident rates of antidepressant fills between the pre-pandemic period and during the COVID-19 pandemic. The counterfactual refers to the predicted values had the pandemic not occurred, and the fitted values are estimated based on the Poisson regression model.

We used ITS (Fig. 1) (Bernal, Cummins, & Gasparrini, 2017, 2020; Bhaskaran, Gasparrini, Hajat, Smeeth, & Armstrong, 2013), a strong quasi-experimental study design (Shadish, Cook, & Campbell, 2002), to compare the monthly incident rate of antidepressant fills from 1 January 2013 to 1 February 2021. The ITS design can identify distinct changes from preexisting trends in antidepressant use, termed a counterfactual. This is instrumental when retrospective evaluations of population-level interventions are undertaken. Full details of the model are provided in the online Supplementary material and Fig. S1.

#### Exposure

We use the term ‘exposure’ to signify the totality of the biopsychosocial adversities associated with the COVID-19 pandemic. This differs from exposure to SARS-CoV-2 virus. The index case of COVID-19 infection in Israel was on 27 February 2020, and the first lockdown started on 14 March 2020. Accordingly, the COVID-19 ‘exposed’ period was defined as the interval between 1 March 2020 and 1 February 2021. The pre-pandemic period from 1 January 2013 to 1 February 2020 was designated as the ‘unexposed’ period. Further details of the COVID-19 pandemic policy restrictions in Israel during the study period are provided in online Supplementary Table S1.

#### Outcome

The primary outcome of the model was based on the incident rate (i.e. new for each person in the sample) of prescribed antidepressant medications purchased (i.e. prescription fills). The date of purchase formed the basis for the analyses. Meuhedet maintains a continuously updated medication registry that includes Anatomical Therapeutic Chemical Classification System codes; code N06A* was used to ascertain antidepressant prescription fills. The antidepressant prescription fill is a measure that is commonly used to infer antidepressant use from administrative data (Cantrell, Eaddy, Shah, Regan, & Sokol, 2006). For brevity, we will refer to this outcome as ‘antidepressant fills’.

#### Covariates

The key covariates were (a) time, modeled as a monthly sequence throughout the study period; (b) exposure to the COVID-19 pandemic; (c) the interaction between time and exposure; (d) an offset term to model event rates; and (e) seasonal Fourier terms to model seasonal components which are oscillations in a time series due to periodic time effects (detail in the online Supplementary material).

### Statistical analysis

The planned data analyses had three aims: (a) to quantify changes in the incident rate of antidepressant fills between the pre-pandemic and the pandemic periods; (b) to examine the robustness of the results by undertaking comprehensive sensitivity analyses; and (c) to forecast incident rates of antidepressant fills beyond the study observation period. Analyses were implemented in R (The R Foundation, 2020), with the packages forecast (Hyndman & Khandakar, 2008; Hyndman et al., 2020) and ggplot2 (Wickham, 2016).

To achieve the first aim, we fitted a Poisson regression model to quantify the association between COVID-19 pandemic exposure and the relative risk of the incident monthly rate of antidepressant fills. The regression model tests for changes in the level and the slope of the incident rate of antidepressant fills for the exposed period, where the level describes the baseline value, and the slope describes the average trend over time. To ascertain the monthly rate of antidepressant prescription fills, the monthly prescription fill count was divided by the monthly number of insured patients. This is done by fitting a Poisson regression model as an offset (additional details in the online Supplementary material). A quasi-Poisson regression model was used to account for the over-dispersion of the data.

To achieve the second aim, we conducted seven different sensitivity analyses: (a) by restricting the primary model to select groups, we tested for the effect of sociodemographic factors and specifically sex (i.e. males, females), age groups (i.e. males of working age, females of working age, persons over 65 years of age), and socioeconomic status (SES) (i.e. low, medium, and high, as defined in the online Supplementary material); (b) we examined the effect of different seasonal decompositions; (c) we used 15-day intervals to test for the possible effect of the time-unit of data aggregation; (d) we examined the effect of lockdown compared to non-lockdown periods during the pandemic (exposed period) to test for the possible impact of severe social restrictions; the specific dates of the lockdown periods are presented in online Supplementary Table S1; (e) we tested for the effect of SARS-CoV-2 status by comparing positive cases to non-cases during the exposure period with a test for equality of proportions (Hogg, Tanis, & Zimmerman, 2013); (f) we repeated the ITS analysis while excluding all cases with serologically confirmed SARS-CoV-2 infection; (g) we compared the incident rate of antidepressant fills during the COVID-19 pandemic with that during the Gaza war to test for the specificity of the findings to the pandemic as opposed to other population-level traumatic periods for Israel. The period of time considered in the analyses is the war months of July and August 2014, and the subsequent month of September 2014.

Our third aim was to extend the primary quasi-Poisson regression model to forecast future incident rate of antidepressant fills at 10 months after the data collection ends (1 March 2021 to 1 December 2021). This period coincides with the uptake of vaccination and the easing of social restrictions. We implemented three forecast scenarios to test for the potential effect of successful vaccination and social restrictions easing, assuming (a) no ongoing effects of the COVID-19 pandemic; (b) ongoing effects of the COVID-19 pandemic; and (c) continuation of the patterns in the periods before and during the COVID-19 pandemic. The first scenario assumes no ongoing effects of the COVID-19 pandemic on antidepressant prescription fills and uses the pre-COVID-19 level and slope (level = −6.2278, slope = −0.0017). The second scenario assumes an ongoing effect of the COVID-19 pandemic on antidepressant prescription fills and uses the post-COVID-19 level and slope (level = −6.2285, slope = 0.0131). The third scenario assumes a continuation of the patterns in the periods before and during the COVID-19 pandemic and uses the pre-COVID-19 slope and the post-COVID-19 level (level = −6.2285, slope = −0.0017).

## Results

### Sample

The sample characteristics at study entry comprised 707 798 individuals (Table 1) and at study end it consisted of 852 233 individuals (male *N* = 424 240, 49.8%; female *N* = 427 993, 50.2%). The total antidepressant fill count was 139 535 (total cumulative rate per 100 000 = 16 372.91, 95% CI 16 287.19–16 459.01) across the entire study period. The monthly incidence of antidepressant fills across the study intervals ranged from 1008.69 to 1845.88, with a mean and standard deviation (s.d.) of 1423.83 (153.29). This represents a range of 118.36 (95% CI 111.20–125.81) to 216.59 (95% CI 206.84–226.60) antidepressant fills per 100 000 in the population.

**Fig. 2.**
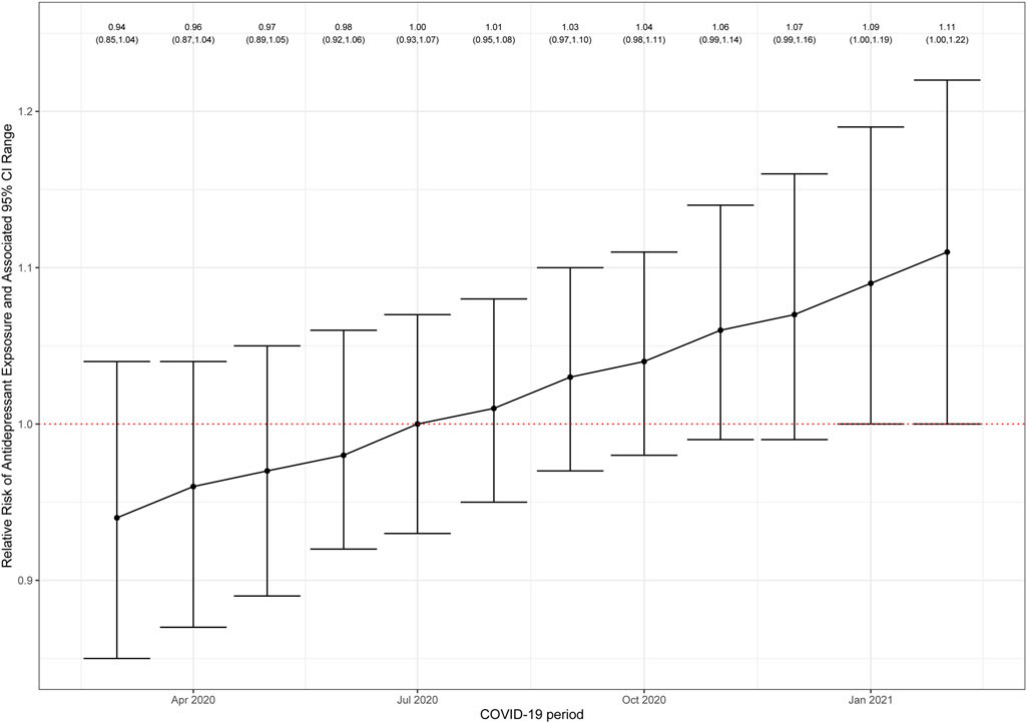
Relative risk of incident rates of antidepressant fills during the COVID-19 pandemic. The relative risk showed a null drop initially and a statistically significant increase during the last two pandemic months examined.

The monthly incidence of antidepressant fills, together with the Poisson regression model fitted values, is shown in Fig. 1 and Table 2. The model assumptions were not violated by residual autocorrelation (online Supplementary Fig. S2). Compared to the counterfactual (i.e. had the COVID-19 pandemic not occurred), the COVID-19 pandemic exposure was associated with an overall 2% increase in monthly antidepressant prescription fills (RR = 1.02, 95% CI 0.96–1.09). In the unexposed period (i.e. pre-pandemic), the incident rate of antidepressant fills showed a progressive, gradual decline. By comparison, the exposed period (i.e. pandemic) was associated with a non-significant initial drop (level change = −0.06, 95% CI −0.16 to 0.04; *p* = 0.24) followed by a steady increase in the incident rate of antidepressant fills (slope change = 0.01, 95% CI 0.00–0.03; *p* = 0.04). This was also reflected in the increase in the RR of monthly antidepressant prescription fills starting from August 2020. The RR was statistically significant in the last 2 months studied (Fig. 2; model details in the online Supplementary material).

**Table 2.** Primary model estimated coefficients, including 95% CIs, *t* statistics, and *p* values

Covariate	Estimate	2.5%	97.5%	*t* statistic	*p* value
Level	−6.2278	−6.2646	−6.1912	−332.61	*p* < 0.001
Slope	−0.0017	−0.0025	−0.0010	−4.70	*p* < 0.001
Level change	−0.0607	−0.1615	0.0386	−1.19	0.24
Slope change	0.0148	0.0007	0.0289	2.05	0.04
Fourier1	0.0525	0.0285	0.0766	4.28	*p* < 0.001
Fourier2	−0.0425	−0.0661	−0.0189	−3.53	*p* < 0.001
Fourier3	−0.0316	−0.0555	−0.0076	−2.59	0.01
Fourier4	0.0293	0.0052	0.0533	2.38	0.02
Fourier5	−0.0408	−0.0578	−0.0238	−4.70	*p* < 0.001

The last five rows correspond to the five significant Fourier terms. The pre-pandemic slope was slightly negative, and the post-pandemic slope switched direction from negative to positive.

### Sensitivity analyses

The key findings of the sensitivity analyses are presented below and in Table 3 and online Supplementary Figs S3–S6.

**Table 3.** Sensitivity analysis of estimated regression coefficients, confidence intervals and *p* values for incident antidepressant fills during the exposure period

Model	Exposure period	
	Level change (95% CI), *p* value	Slope change (95% CI), *p* value
Primary model	−0.06 (−0.16 to 0.04) 0.24	0.01 (0.00–0.03) 0.04
Males	−0.07 (−0.18 to 0.03) 0.18	0.01 (0.00–0.02) 0.19
Females	−0.05 (−0.17 to 0.06) 0.36	0.02 (0.00–0.03) 0.02
Working-age males	−0.10 (−0.22 to 0.02) 0.10	0.02 (0.00–0.03) 0.07
Working-age females	−0.05 (−0.17 to 0.07) 0.43	0.02 (0.00–0.04) 0.02
Young adult males	−0.12 (−0.25 to 0.01) 0.09	0.02 (0.00–0.04) 0.05
Young adult females	−0.07 (−0.20 to 0.06) 0.29	0.02 (0.01–0.04) 0.01
Middle aged males	−0.07 (−0.21 to 0.07) 0.35	0.01 (−0.01 to 0.03) 0.32
Middle aged females	−0.02 (−0.16 to 0.12) 0.82	0.01 (−0.01 to 0.03) 0.23
Senior males	0.05 (−0.09 to 0.18) 0.48	−0.01 (−0.03 to 0.01) 0.16
Senior females	−0.06 (−0.21 to 0.08) 0.40	0.01 (−0.01 to 0.04) 0.16
Low SES	−0.04 (−0.22 to 0.14) 0.68	0.02 (0.00–0.05) 0.08
Medium SES	−0.08 (−0.19 to 0.03) 0.15	0.01 (0.00–0.03) 0.09
High SES	0.00 (−0.13 to 0.13) 0.98	0.00 (−0.01 to 0.02) 0.62
⩾65 years	−0.01 (−0.13 to 0.10) 0.81	0.00 (−0.01 to 0.02) 0.76
Loess STL	−0.07 (−0.16 to 0.02) 0.16	0.02 (0.00–0.03) 0.02
Moving average	−0.07 (−0.17 to 0.02) 0.15	0.02 (0.00–0.03) 0.02
15-day aggregation	−0.06 (−0.15 to 0.03) 0.21	0.01 (0.00–0.01) 0.03
Gaza war	−0.06 (−0.16 to 0.04) 0.23	0.01 (0.00–0.03) 0.04
Gaza war males	−0.07 (−0.18 to 0.03) 0.17	0.01 (0.00–0.03) 0.18
Gaza war females	−0.05 (−0.17 to 0.06) 0.36	0.02 (0.00–0.03) 0.02
Lockdown	0.00 (−0.12 to 0.11) 0.98	0.00 (−0.01 to 0.01) 0.56
No COVID-19 cases	−0.06 (−0.16 to 0.04) 0.23	0.02 (0.00–0.03) 0.02

SES, socioeconomic status; STL, seasonal and trend decomposition; Loess and moving averages are both types of seasonal decompositions.

#### Sociodemographic features

The statistically significant increase in the slope of the incident rate of antidepressant fills was replicated in the analyses restricted to females (Table 3, online Supplementary Fig. S3), and females of working age (online Supplementary Fig. S4). During the entire study period, the monthly rate of antidepressant prescription fills for women ranged from 127.61 (95% CI 117.32–138.74) to 241.96 (95% CI 227.54–257.02) per 100 000 in the population, whereas the monthly rate of antidepressant prescription fills for men ranged from 106.34 (95% CI 96.95–116.59) to 191.01 (95% CI 178.23–204.54). The pre-pandemic antidepressant prescription fill rates for men were almost constant (with seasonal fluctuations), while the pre-pandemic rates were decreasing for women (online Supplementary Fig. S3). During the pandemic the rate of increase was much larger in women than in men, with a slope change almost double in size for women compared to men (Table 3, online Supplementary Fig. S3).

No change in the slope in analyses restricted to all males (Table 3, online Supplementary Fig. S3), males of working age (Table 3, online Supplementary Fig. S4), different SES groups (Table 3, online Supplementary Fig. S5), and among persons aged 65 years and older (Table 3, Supplementary Fig. S6). Follow-up analyses were conducted to further explore the sex-related findings. These sensitivity analyses involved young (aged 15–44 years) males and females, middle-aged males and females (aged 45–64 years), and senior (aged over 65 years) males and females. The statistically significant slope change replicated among young females (Table 3, online Supplementary Figs S5–S7).

#### Seasonal components

Seasonal decomposition replicated the findings of the primary model (Table 3, online Supplementary Fig. S7).

#### 15-day Aggregates

Altering the time-unit for data aggregation from a month to 15-day intervals replicated the findings of the primary model (Table 3, online Supplementary Fig. S8).

#### Lockdown on/off periods

Addition of the lockdown periods nullified the effect of the pandemic on the slope of the incident rate of antidepressant fills (Table 3, Supplementary Fig. S9).

#### SARS-CoV-2 status

The proportion of incident antidepressant fills counts during the exposure period was significantly higher among SARS-CoV-2-positive cases compared to non-cases [difference = 10^−5^ (−137.89), 95% CI 10^−5^ (−149.81 to −125.96), *p* < 0.001], with a very small effect size (Cohen's *h* = 0.046, 95% CI 0.040–0.052). A follow-up sensitivity analysis restricted only to SARS-CoV-2 non-cases yielded identical point-precision estimates to the primary analysis (Table 3, online Supplementary Fig. S10).

#### Negative control

The Gaza war was associated with a non-statistically significant level and slope change (Gaza war level change = −0.0347, 95% CI −0.1955 to 0.1201; *p* = 0.67, Gaza war slope change = 0.0491, 95% CI −0.0724 to 0.1707; *p* = 0.43). Comparison of the incident rates of antidepressant fills between the pandemic and the Gaza war yielded showed a higher slope during the COVID-19 pandemic (Table 3, online Supplementary Table S3, Fig. S15).

### Forecasting

**Fig. 3.**
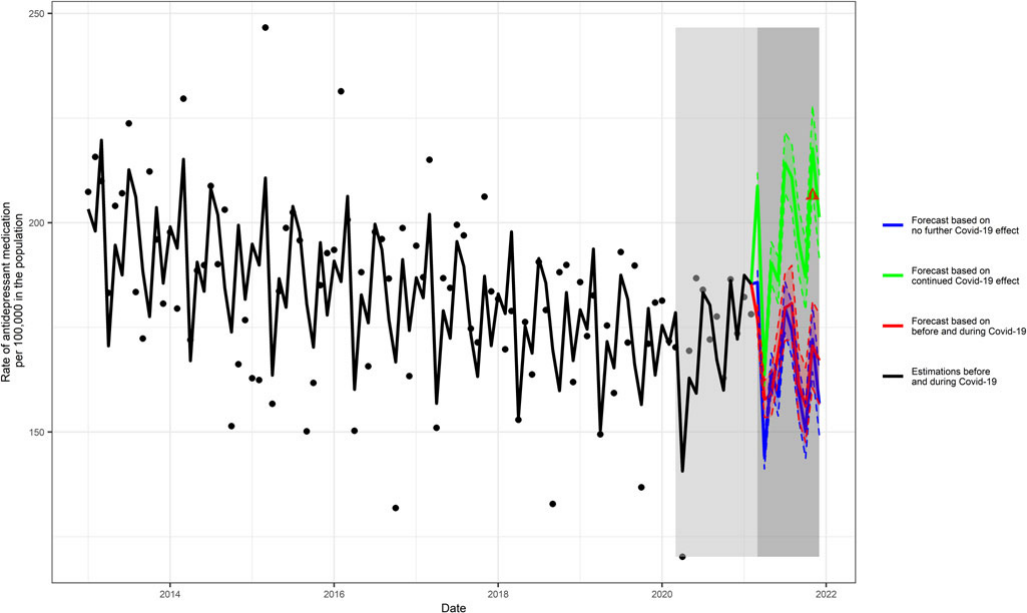
Forecasted 10-month incident antidepressant fills. Forecasted values are presented for three scenarios: (1) assuming no ongoing effects of the COVID-19 pandemic due to the successful vaccination roll-out; (2) assuming ongoing effects of the COVID-19 pandemic despite the successful vaccination; and (3) based on the intervals before and during COVID-19 pandemic. The light gray rectangle represents the observed COVID-19 period, and the dark gray rectangle represents the forecast period. The actual value in November 2021 is shown in the red triangle.

Three forecasting scenarios for a 10-month period were considered (1 March 2021 to 1 December 2021) (online Supplementary Table S2, Fig. S11). Multi-step prediction intervals were calculated using the multi-step forecast standard deviation for the drift method (Hyndman & Athanasopoulos, 2018). The median incident rate of antidepressant fills in the unexposed period (i.e. pre-pandemic) was 184.04. The predicted incident rate of antidepressant fills was 156.87, 95% PI (149.38–164.73), assuming no ongoing pandemic effect (scenario 1) and 201.32, 95% PI (191.72–211.42) assuming an ongoing pandemic effect (scenario 2). The third scenario was based on the periods before and during the pandemic and yielded a predicted antidepressant fill rate of 167.11 (95% PI 156.69–178.22). The actual incident rate of antidepressant fills in November 2021 was 206.48 (95% CI 197.11–216.27), which is consistent with the scenario that assumes an on-going pandemic effect (Fig. 3).

## Discussion

Using a quasi-experimental design, nationwide data, and robust sensitivity analyses, we observed an increased incident rate of antidepressant fills during 11 months of the COVID-19 pandemic in Israel, compared to pre-pandemic rates. This trend was not attenuated by lockdown periods, SES, or SARS-CoV-2 infection, and was more pronounced in women than men. Moreover, there was greater demand for antidepressants during COVID-19 than during a comparison to a wartime period. These findings did not reflect methodological artefacts of seasonal trends and were not influenced by the length of data aggregation intervals.

The change in the rate of antidepressant prescription fills at the beginning of the COVID-19 pandemic was not sudden, but a switch from a very subtle decreasing trend before the pandemic, to an increasing trend following the pandemic. Namely, the COVID-19 pandemic was not associated with an immediate increase in antidepressant prescription fills, but it was associated with an increase over time and is expected to continue increasing in the near future. The increased incident rate of antidepressant fills during the pandemic in Israel is consistent with prior reports that used nationwide data from the Europe and the USA (Estrela et al., 2022; Express Scripts, 2020; Rabeea et al., 2021) and aligns with similar findings following other events producing population-level distress, such as terrorist attacks (Druss & Marcus, 2004) and earthquakes (Trifirò et al., 2013). The increased antidepressant fill rate during the pandemic was higher than that seen during the Gaza war, probably reflecting the greater length, morbidity, and mortality associated with the pandemic.

The data presented here suggest that the increase in antidepressant use during the pandemic was more pronounced in women. This finding is consistent with evidence from multiple nations that indicates that the toll of the COVID-19 pandemic has been greater on the mental health of women than men (COVID-19 Mental Disorders Collaborators, 2021; Lindau et al., 2021; NCHS, 2021; ONS, 2021b; Statistics Canada, 2021). For instance, nationwide studies from the USA (NCHS, 2021) and the UK (ONS, 2021b) report that approximately 10% more women than men experienced moderate to severe depressive symptoms during the pandemic. There are several plausible mechanisms that may explain the COVID-19 impact on women such as greater vulnerability of women to depression/anxiety, greater exposure to adversity, or sex differences in help-seeking behavior. The current study design does not permit causative inferences about these sex differences, but a range of reasons has been proposed. Among these are greater caregiving burden and domestic violence (Kofman & Garfin, 2020), and higher drop-out from the workplace among women than men (United Nations, 2020). However, it is important to consider the possible role of the coincident shift of mental health care provision from traditional in-person settings to telehealth modalities (telephone or video). Data on the pattern of consultations with general practitioners in the UK suggest a more significant drop in appointments for men than women throughout the pandemic (NHS Digital, 2021), including consultations for mental health problems (ONS, 2021c).

We observed no change in the incident rates of antidepressant fills when analyses were restricted to those above the age of 65 years. Several explanations may be postulated including the drop in medical consultations in this age group during the pandemic for all health problems, including mental health (e.g. Hirschtritt, Slama, Sterling, Olfson, & Iturralde, 2021; ONS, 2021c), despite the significant increase in mental health problems reported by older individuals (Tyler et al., 2021). For individuals aged 65 years or older, difficulties accessing telehealth may act as a substantial barrier (Lam, Lu, Shi, & Covinsky, 2020) because of unfamiliarity with the relevant technology.

Sensitivity analyses stratified by SES identified a null gradient of lower incident rate of antidepressant fills the higher the SES. The lack of marked SES differences in the incident rate of antidepressant fills is likely to reflect the wide availability of services to the Israeli population, which is legislatively mandated, and the widespread effect of the pandemic that transcended SES to some extent.

Sensitivity analyses of the effect of three lockdowns did not identify any changes in incident rate of antidepressant fills that mirrored the on/off periods of the imposed social restrictions. Similar findings regarding pharmacy claims for antidepressant prescriptions have been reported both in the UK and the USA (Express Scripts, 2020; Rabeea et al., 2021). Collectively, this evidence suggests that psychological distress, and associated help-seeking, remained elevated throughout the pandemic and were not restricted to the lockdown periods.

The increased incident antidepressant fill rate was not driven by for SARS-CoV-2-positive cases as shown by the results of the sensitivity analyses restricted to SARS-CoV-2 non-cases. Also, the proportion of antidepressant prescriptions issued to individuals with documented SARS-CoV-2 infections was significantly higher than that in non-cases but with a very small effect size. This is in line with prior studies reporting increased level of mental health problems in SARS-CoV-2 survivors that notably include anxiety and depression (Mazza et al., 2020; Xie, Liu, Xu, & Zhong, 2021).

Our forecasting analyses examined three possible scenarios. The first scenario assumes that COVID-19 will no longer affect antidepressant prescription fills and the rate of antidepressants fills will decrease. The second scenario assumes that COVID-19 will continue having the same effect on antidepressant prescription fills and uses the post-COVID-19 level and slope. This scenario predicts the post-COVID-19 trend in antidepressant fills will continue upwards. The third scenario uses the pre-COVID-19 slope and the post-COVID-19 level and predicts that the rate of antidepressant fill will decrease but starting from the marginally lower post-COVID-19 level. The scenario that assumed a continued effect of COVID-19 predicted an incident rate of 201.32 incident antidepressant fills. Data from Meuhedet for the month of November 2021 indicate that the observed rate, which was 206.48, is very close to that predicted under the assumption that the successful vaccination roll-out in Israel would do little to curb pandemic-related distress. These findings are not surprising given ongoing concerns about breakthrough infections and persistent disruption in social and economic life in Israel. The accuracy of the forecast strengthens our belief in the adequacy of the Poisson regression model and the chosen covariates. We note that forecast scenarios based on 8 years of prior data such as those used here are valid for up to 2 years and their accuracy is less reliable thereafter as prediction intervals become wider and wider. The forecast scenarios cannot fully account for all the possible effects of the pandemic, which are themselves difficult to predict, and possible significant changes to health policy which might influence antidepressant prescription fills in the future.

The findings of this study add to the general evidence for the toll exerted by the COVID-19 on population mental health. The study captures a single aspect of the response to the pandemic-induced psychological distress as inferred by increased antidepressant use. Our data do not allow us to comment on the appropriateness of the prescribed medication, its efficacy, or the possible long-term effects of increased antidepressant use for population health. The response to the pandemic mental health crisis has varied amongst countries but the most common adaptation has been to develop or enhance digital and phone-based delivery of health information and health care (OECD, 2021). However, specific plans for maintaining and improving response to the current mental health problems or for improving preparedness for future pandemics remain unclear but emphasize a whole-society approach to recovery and resilience that extends beyond mental health services to school- and work-based psychological support (OECS, 2022; Regional Health-Europe TL, 2022).

The study has several limitations. First, we did not match prescriptions to diagnosis. Accordingly, it is not possible to link any specific indication in the observed increase in antidepressant consumption. Second, we are unable to comment on the severity of the health problems reported in the consultations that led to an antidepressant prescription, on the number of mental health problems that may have been missed during consultations leading to under-prescribing on antidepressants and on the number of individuals that decided not to purchase antidepressants even when these were prescribed. Third, we implemented an interrupted time-series study that is a quasi-experimental study design (Shadish et al., 2002). A stronger quasi-experimental design, namely the parallel control group that has a control group unexposed, was unavailable, and it was not possible to eliminate all possible sources of residual confounding (e.g. minority groups at increased risk of depression). These limitations make causal inference difficult in observational data such as used in this study. Fourth, our analyses were limited to one class of psychotropics (i.e. antidepressants) but there is some evidence that prescriptions of all psychotropics may have increased during the pandemic (Express Scripts, 2020; Hirschtritt et al., 2021). Further, we did not collect information on the different types of antidepressants prescribed or other forms of treatment such as psychotherapy.

In sum, harnessing the joint strengths of a quasi-experimental study design and a nationally representative data from Israel, our results underscore the significant impact of COVID-19 on women's mental health and the ongoing risk caused by the enduring social and economic repercussions of the COVID-19 pandemic. Both findings offer critical information to mental health policy and service delivery in the aftermath of the pandemic restrictions.

## Supplementary material

For supplementary material accompanying this paper visit https://doi.org/10.1017/S0033291722001891. click here to view supplementary material
