# Supplementary material for: Increased incident rates of antidepressant use during the COVID-19 pandemic: interrupted time-series analysis of a nationally representative sample
Source: Psychol Med. 2022 Jun 10;53(11):4943–51. doi: 10.1017/S0033291722001891 (PMC9273730; doi:10.1017/S0033291722001891)
Supplement: Supplementary file 1 [file S0033291722001891sup001.docx]

**Increased Incident rates of antidepressant use during the COVID-19 pandemic: Interrupted time series analysis of a nationally representative sample**

**Supplementary Material**

**1. Model details**

**2. Definition of socioeconomic status**

**3. Supplemental Table 1. Public health restrictions during the pandemic in israel**

**4. Definition of sars-cov-2 status**

**5. Supplemental Figure 1. Scatter plot of the monthly rate of antidepressant fills**

**during the study period**

**6. Supplemental Figure 2: Residual autocorrelation in the primary poisson model**

**7. Supplemental Figure 3. Scatterplot and regression fitted values by sex**

**8. Supplemental Figure 4. Scatterplot and regression fitted values by working age by**

**sex**

**9. Supplemental Figure 5. Scatterplot and regression fitted values for young adults**

**by sex**

**10. Supplemental Figure 6 Scatterplot and regression fitted values for middle aged**

**males and females**

**11. supplemental figure 7. Scatterplot and regression fitted values for senior males**

**and females**

**12. supplemental figure 8 Scatterplot and regression fitted values for different ses**

**groups**

**13. supplemental figure 9. Scatterplot and regression fitted values for adults aged**

**over 65 years**

**14. Supplemental Figure 10. Scatterplot and regression fitted values for stl and ma**

**seasonal decompositions**

**15. Supplemental Figure 11. Scatterplot and regression fitted values aggregated in**

**15 day intervals**

**16. Supplemental Figure 12. Scatterplot and regression fitted values for 15 day**

**intervals and lockdown periods**

**17. Supplemental Figure 13. Scatterplot and regression fitted values restricted to**

**Sars-Cov-2 non-cases**

**18. Supplemental Figure 14. Scatterplot and regression fitted values for the Covid-**

**19 pandemic and 2014 gaza war**

**19. Supplemental Figure 15. Scatterplot and regression fitted values for the Covid-**

**19 pandemic and 2014 gaza war by sex**

**20. Supplemental Table 2. Forecasted values and 95% prediction intervals for the**

**three scenarios**

**21. Supplemental Table 3. Complete model summary for the gaza war**

**22. Supplemental References**

**Supplementary Methods**

# Model Details

We fitted the monthly antidepressant medications fill ($Y_{t}$) using a Poisson regression model with the following covariates: time ($t$), exposure (classified as unexposed or exposed) as a binary exposure ($X_{t}$), the exposure interaction with time (${t\cdot X}_{t}$), and seasonal components ($S_{t}$). A Poisson regression model is a special case of a generalized linear regression model (GLM). In a GLM we model a function of the conditional expectation, denoted by $g(E\left[ Y_{t} | {t, X}_{t}, S_{t} \right])$, as a linear function of its covariates. The function $g\left( \cdot\right)$ is known as a link function, and different GLMs use different link functions. A Poisson regression model uses the log link function $g\left( \cdot\right)=log(\cdot)$. As we are modeling event rates, we also add an offset term (log(N_t )) to the Poisson regression model, where N_t is the monthly number of insured patients. The multivariate Fourier terms are denoted by S_t, and their vector of coefficients is denoted by γ. Hence, $\gamma^{T}S_{t}$is the contribution of the Fourier terms to the regression model, where the superscript T denotes the transpose operation (dot product between two vectors).

A Poisson regression model assumes that the variance of the outcome (prescription fills) is equal to its mean. Overdispersion occurs when the variance of the outcome is greater than the mean, in which case a quasi-Poisson model should be used to account for this overdispersion. A quasi-Poisson model assumes that the variance is a linear function of the mean, and includes estimation of this linear weight parameter. This parameter is also known as the dispersion parameter.

As the variance of the monthly prescription fills is considerably larger than its mean, we used a quasi-Poison regression model to account for this over-dispersion of the data. Note that we added an offset term to the regression model since it is appropriate to focus on the rate of antidepressant fill and not the count itself. The monthly rate of antidepressant prescription fills is obtained by dividing the monthly count of antidepressant prescriptions fills with the monthly number of insured patients; the logarithm of the monthly number of insured patients defines the offset term in our regression model. Additionally, we used Fourier terms to model the seasonal component (Bernal, Cummins, & Gasparrini, 2017). Seasonal components are periodic oscillations in a time series that are due to the periodic time effect. For our analysis, we chose the five Fourier terms that were statistically significant (P<0.05) in the quasi-Poisson model. The seasonal pattern was estimated based on data up to February 2020, not including the Covid-19 period. Our Poisson regression model can be presented using the following notation; denote by $t$ the time (a sequence of months from 1 to 98, with $t=87$ for March 2020), by $Y_{t}$ the monthly antidepressant medications count, by $X_{t}=\mathbf{1}_{\{t\geq87\}}$ the Covid-19 indicator, by $N_{t}$ the monthly number of insured patients, and by $S_{t}$ the seasonal component (multivariate Fourier terms). Then our regression model is

$$\text{log}\left( E\left( Y_{t}\mid N_{t}, X_{t},t,S_{t} \right) \right)=\text{log}\left( N_{t} \right)+\beta_{0}+\beta_{1}\cdot t+\beta_{2}\cdot X_{t}+\beta_{3}\cdot tX_{t}+\gamma^{T}S_{t},$$

where $\beta_{0}$ is the intercept, $\beta_{1}$ is the time trend coefficient, $\beta_{2}$ is the level change following the intervention, $\beta_{3}$ is the slope change following the intervention, and $\gamma\in\mathbb{R}^{5}$ is the multivariate coefficient of the Fourier terms.

To quantify further the effect size of the Covid-19 period on antidepressant prescription fills, we offer a formula to quantify the relative risk (RR). First, we calculate the monthly RR for each of the time points in the Covid-19 period, and then use a geometric mean to obtain the final average RR for the entire Covid-19 period. We estimate the monthly RR at time $t$, $t^{*}\leq t\leq n$ by

$$\text{RR}\left( t \right)=\frac{\text{exp}\left( \hat{\beta_{0}}+\hat{\beta_{1}}\cdot t+\hat{\beta_{2}}+\hat{\beta_{3}}\cdot\left( t-t^{*} \right)+\hat{\gamma}^{T}S_{t} \right)}{\text{exp}\left( \hat{\beta_{0}}+\hat{\beta_{1}}\cdot t+\hat{\gamma}^{T}S_{t} \right)}=\text{exp}\left( \hat{\beta_{2}}+\hat{\beta_{3}}\cdot(t-t^{*}) \right),$$

where we compare the model predictions for the Covid-19 period (in the numerator), with their model-based counterfactual values (in the denominator). The model-based counterfactual values are the model predictions for the Covid-19 period assuming that Covid-19 did not occur, and so continue the existing trend based on the pre-Covid-19 period. Finally, the average RR estimate for the entire Covid-19 period can now be calculated using a geometric mean

$$\text{RR}=\left( \prod_{t=t^{*}}^{n} \text{RR}\left( t \right) \right)^{\frac{1}{n-t^{*}+1}}=\text{exp}\left( \hat{\beta_{2}}+\frac{n-t^{*}}{2}\cdot\hat{\beta_{3}} \right)$$

where the last equality follows from exponentiation rules.

As both the monthly RR and the average RR depend on the level change and the slope change of the quasi-Poisson regression model. As a result, we can calculate confidence intervals for these RRs that are based on the variances and covariances of the regression coefficients from the quasi-Poisson regression model.

Three possible forecast scenarios were based on this model:

The first scenario assumes no ongoing effects of the COVID-19 pandemic on antidepressant prescription fills, and uses the pre-Covid-19 level and slope (level=$\beta_{0}$and slope= $\beta_{1}$).

The second scenario assumes an ongoing effect of the COVID-19 pandemic on antidepressant prescription fills, and uses the post-Covid-19 level and slope (level=$\beta_{0}+\beta_{2}$and slope= $\beta_{1}+\beta_{3}$).

The third scenario assumes a continuation of the patterns in the periods before and during the COVID-19 pandemic, and uses the pre-Covid-19 slope ($\beta_{1}$) and the post-Covid-19 level ($\beta_{0}+\beta_{2}$).

# Definition of Socioeconomic Status

Socioeconomic status was ascertained using the Central Bureau of Statistics (CBS) Registry. This registry enables the identification of each person’s residential area (i.e., a neighborhood measure of socioeconomic status; SES) and link data to socioeconomic measures from the household census data. This method of SES assignment has been widely used in research (Levine, Levav, Yoffe, Becher, & Pugachova 2016).

.

# Supplemental Table 1. Dates of Public Health Restriction During the Pandemic and descriptions

|  | | | |
| --- | --- | --- | --- |
|  | **Date begins**  **YYY-MM-DD** | **Date ends**  **YYY-MM-DD** | **Restrictions** |
| Pre-lockdown 1 | 2020-03-01 | 2020-03-13 | International travel banned |
| Lockdown 1 | 2020-03-14 | 2020-04-30 | All schools and workplaces closed, restrictions on gatherings, stay at home, internal movement restrictions, international travel banned |
| Post-lockdown 1 | 2020-05-01 | 2020-09-18 | Restrictions on gatherings, internal movement restrictions, international travel banned |
| Lockdown 2 | 2020-09-18 | 2020-10-17 | All schools and workplaces closed, restrictions on gatherings, stay at home, internal movement restrictions |
| Post-lockdown 2 | 2020-10-18 | 2020-12-26 | All schools closed, restrictions on gatherings, internal movement restrictions, international travel banned |
| Lockdown 3 | 2020-12-27 | 2021-02-07 | All schools and workplaces closed, restrictions on gatherings, stay at home, internal movement restrictions, international travel banned |
| Entire COVID-19 period | 2020-03-01 | 2021-02-28 | Public events canceled |

All schools closed except nurseries. Workplaces closed for all but essential workplaces. For each interval and each restriction, we compared the mode value of the restriction to a threshold to classify whether the restriction occurred during the interval. For most restrictions, we used the Oxford COVID-19 Government Response Tracker threshold value of two, while we used a threshold value of three for international travel ban, restrictions on gatherings, and workplaces closures (Hale et al., 2021).

# Definition of SARS-CoV-2 Status

The positive cases were identified based on serological testing for antigen identification as provide in the electronic health records. Study participants with either negative serological tests or participants with no relevant tests and no clinical symptoms were designated as non-cases.

# Supplemental Figure 1. Scatter plot of the monthly rate of antidepressant fills during the study period


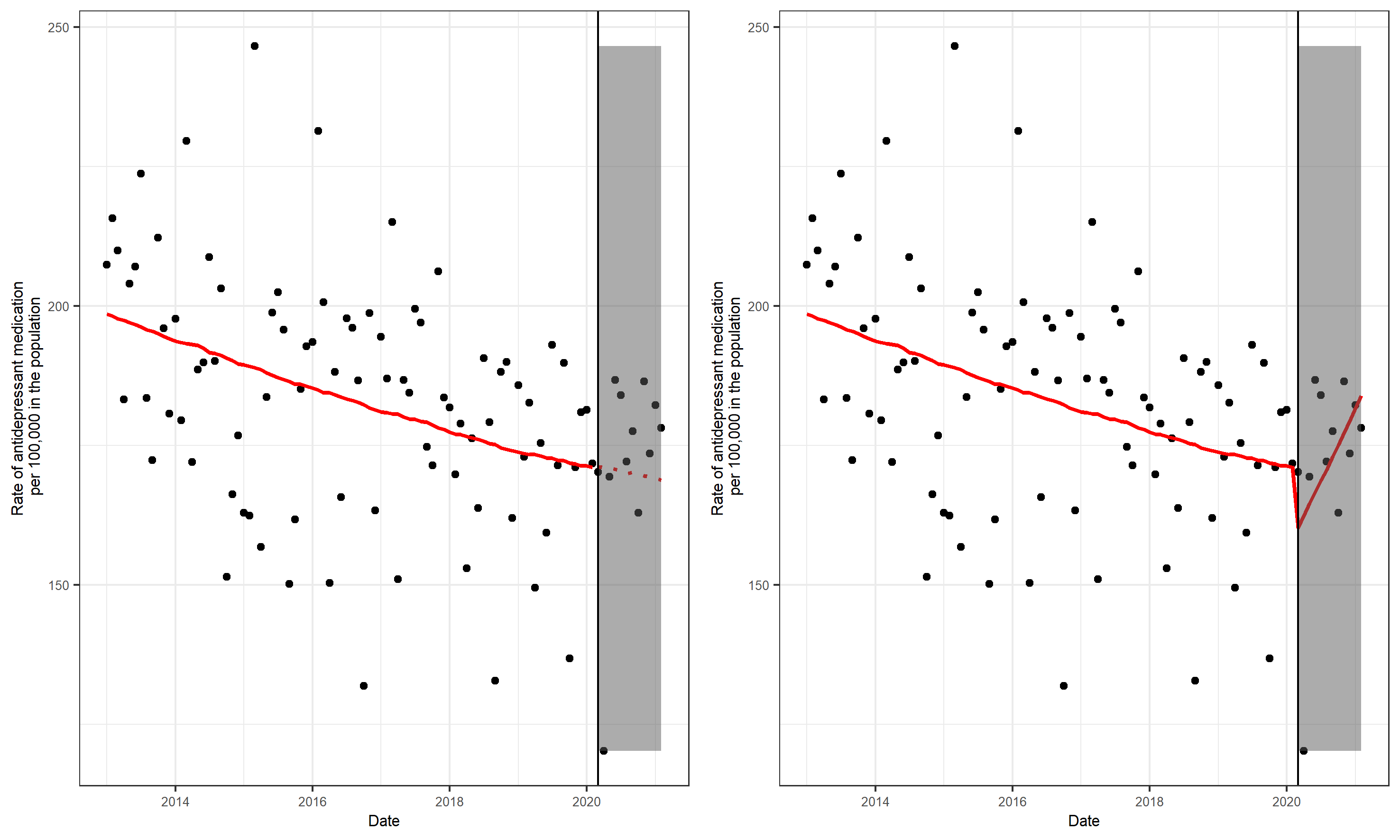


**Supplemental Figure 1.** **Scatter Plot of the Monthly Rate of Antidepressant Fills During the Study Period.** White background: pre-COVID-19 period, grey background: post-COVID-19 period. Left: The red continuous line is the fitted pre-COVID-19 trend, and the dotted red line is the predicted trend had the COVID-19 pandemic not occurred (couterfactual). Right: The red continuous line is the fitted pre and post COVID-19 trend.

# Supplemental Figure 2: Residual autocorrelation in the primary poisson model
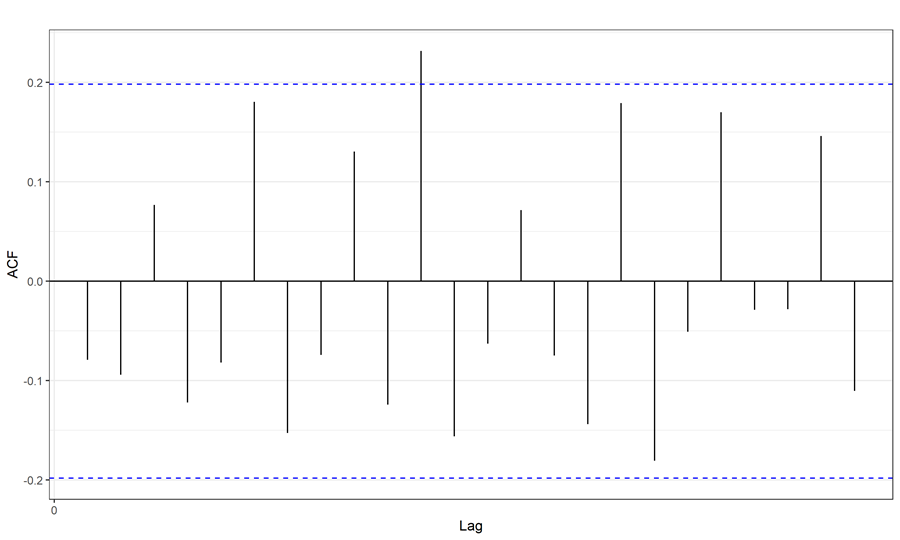


**Supplementary Figure 2: Residual Autocorrelation in the Primary Poisson Model.** The blue dashed lines represent a 95% confidence interval. Any correlation within the blue dashed lines is not considered different than zero. In this case we observe that for all but one of the ACFs are within the confidence interval, and hence do not violate the white noise assumption stating the residuals are uncorrelated. ACF=autocorrelation function

# Supplemental Figure 3. Scatterplot and regression fitted values by sex


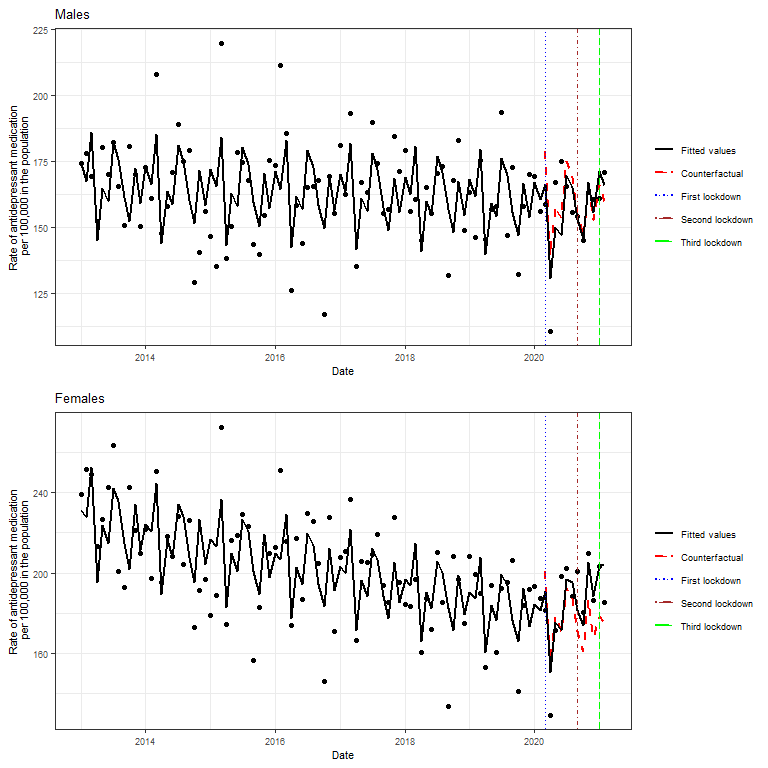


**Supplemental Figure 3. Scatterplot and Regression Fitted Values by Sex**. Top panel: males; Lower panel: females. Scatter plot of the monthly rate of incident antidepressant fills. Black lines: regression fitted values; Red lines: counterfactual. The horizontal lines marked the onset of the first (blue), second (brown) and third lockdown (green) in Israel. Please see Table 2 for statistical values.

# Supplemental Figure 4. Scatterplot and regression fitted values by working age by sex


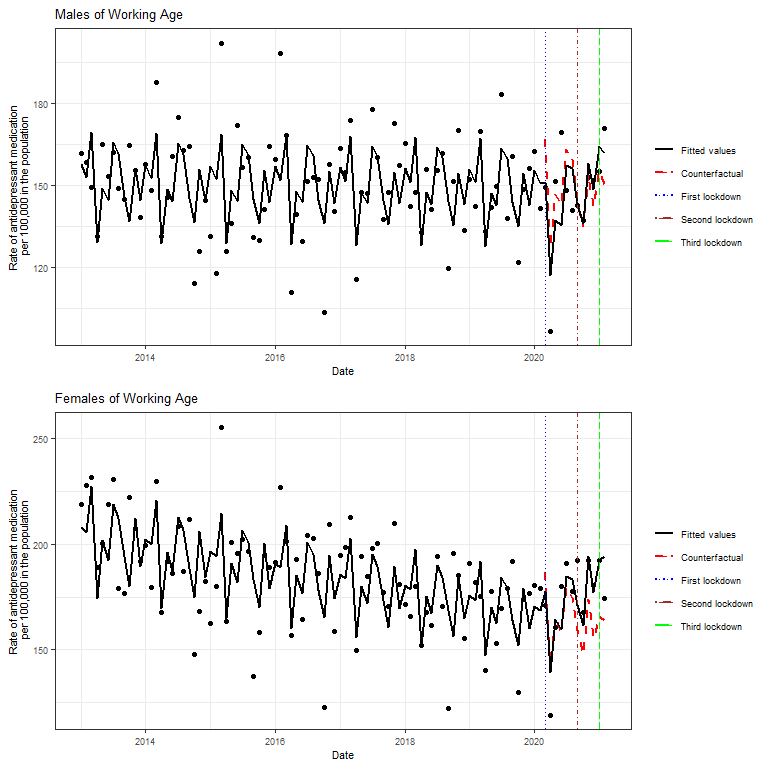


**Supplemental Figure 4. Scatterplot and Regression Fitted Values for Males of Working Age and Females of Working Age**. Top panel: males; Lower panel: females. Scatter plot of the monthly rate of incident antidepressant fills. Black lines: regression fitted values; Red lines: counterfactual. The horizontal lines marked the onset of the first (blue), second (brown) and third lockdown (green) in Israel. Please see Table 2 for statistical values.

# Supplemental Figure 5. Scatterplot and regression fitted values for young adults by sex


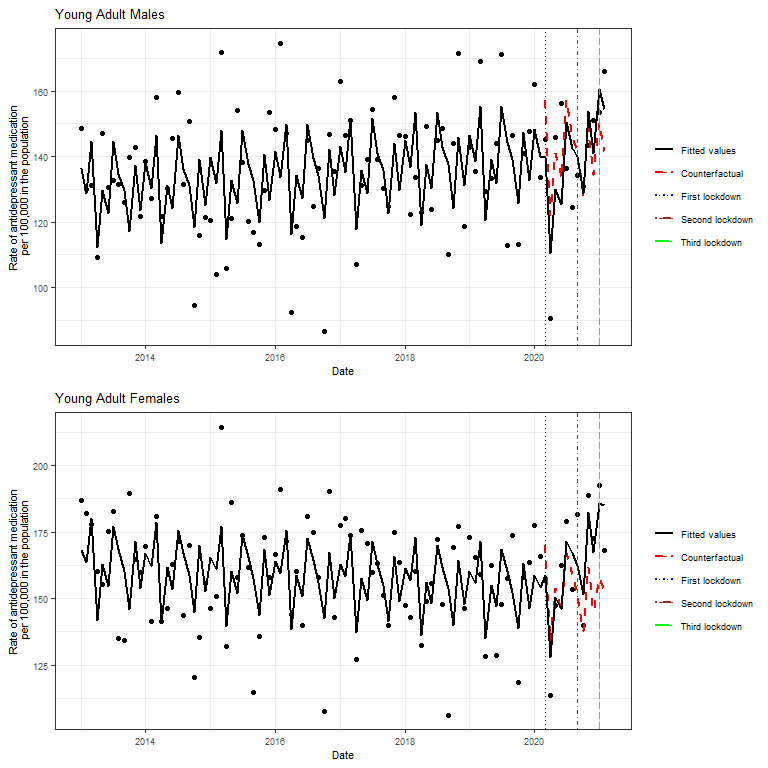


**Supplemental Figure 5. Scatterplot and Regression Fitted Values for Young Adult aged 15-44 Males and Females**. Top panel: males; Lower panel: females. Scatter plot of the monthly rate of incident antidepressant fills. Black lines: regression fitted values; Red lines: counterfactual. The horizontal lines marked the onset of the first (blue), second (brown) and third lockdown (green) in Israel. Please see Table 2 for statistical values..

# Supplemental Figure 6 Scatterplot and regression fitted values for middle aged males and females


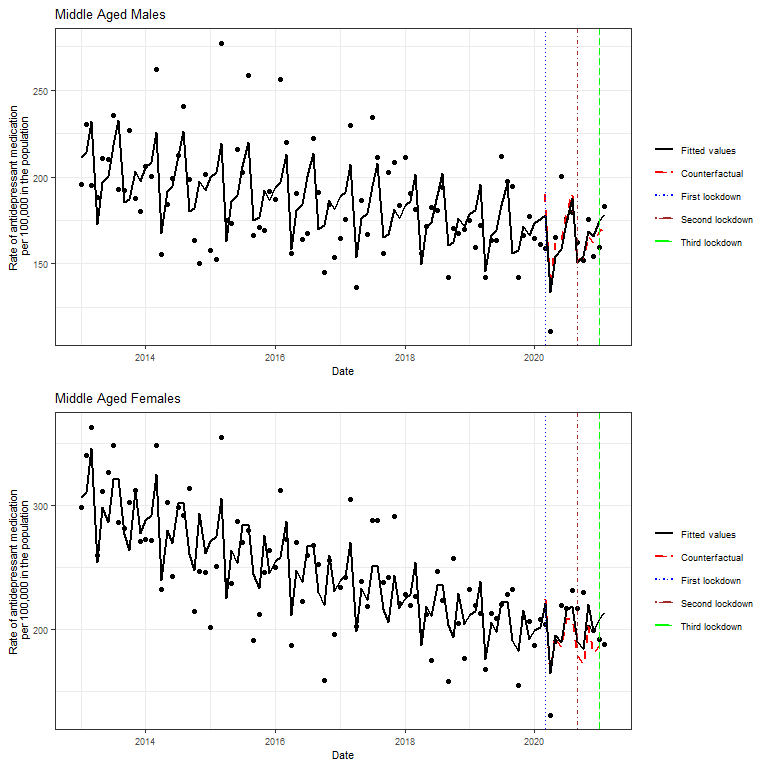
 **Supplemental Figure 6. Scatterplot and Regression Fitted Values for Middle-Aged (45-64) Males and Females**. Top panel: males; Lower panel: females. Scatter plot of the monthly rate of incident antidepressant fills. Black lines: regression fitted values; Red lines: counterfactual. The horizontal lines marked the onset of the first (blue), second (brown) and third lockdown (green) in Israel. Please see Table 2 for statistical values.

# Supplemental Figure 7. Scatterplot and regression fitted values for senior males and females


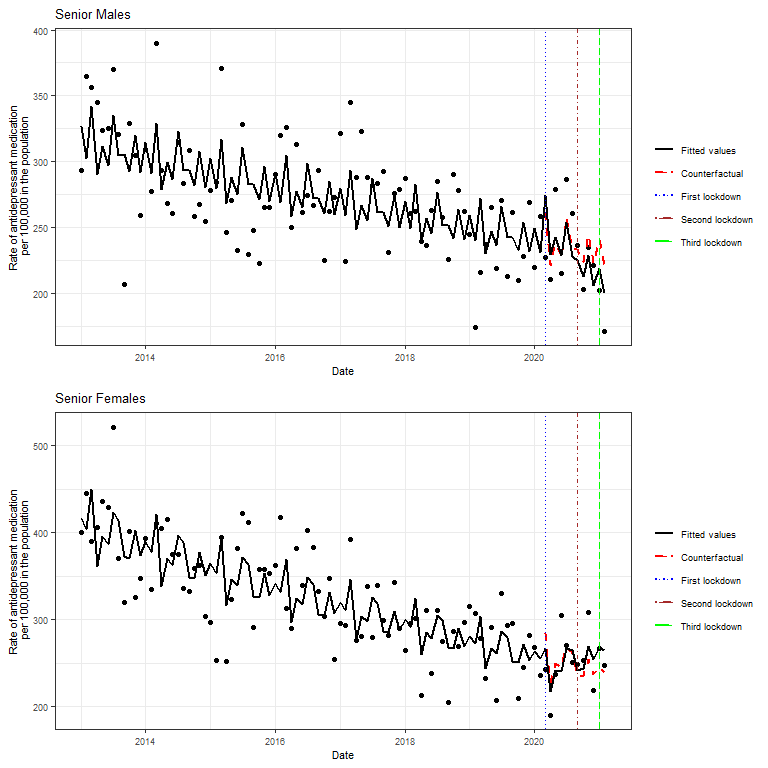
**Supplemental Figure 7. Scatterplot and Regression Fitted Values for Senior (aged 65+) Males and Females**. Top panel: males; Lower panel: females. Scatter plot of the monthly rate of incident antidepressant fills. Black lines: regression fitted values; Red lines: counterfactual. The horizontal lines marked the onset of the first (blue), second (brown) and third lockdown (green) in Israel. Please see Table 2 for statistical values..

# Supplemental Figure 8 Scatterplot and regression fitted values for different ses groups


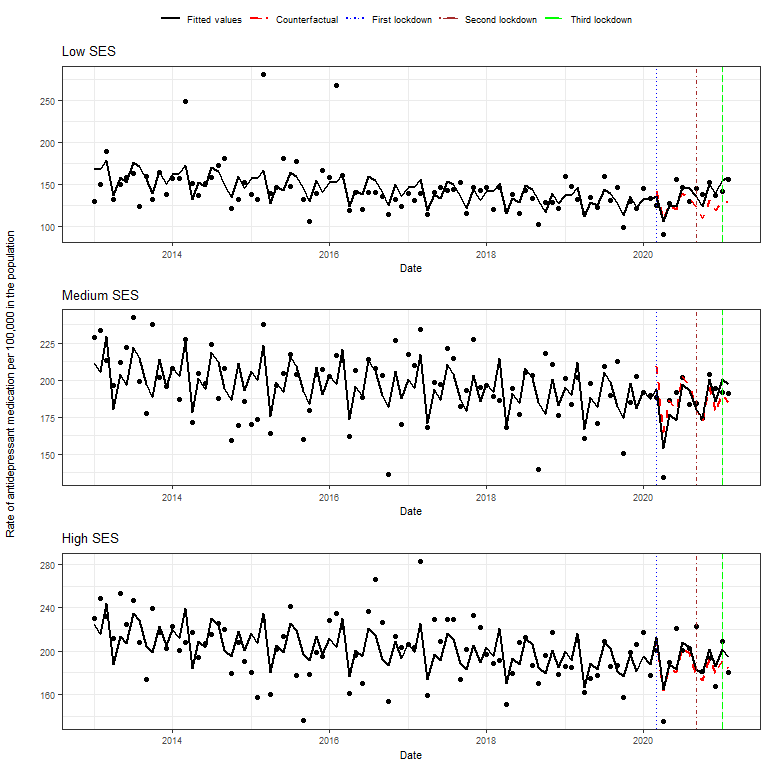


**Supplemental Figure 8. Scatterplot and Regression Fitted Values for Different SES Groups.** Top panel: Low SES; Middle panel: middle SES; Lower panel: high SES. Scatter plot of the monthly rate of incident antidepressant fills. Black lines: regression fitted values; Red lines: counterfactual. The horizontal lines marked the onset of the first (blue), second (brown) and third lockdown (green) in Israel. Please see Table 2 for statistical values..

# Supplemental Figure 9. Scatterplot and regression fitted values for adults aged over 65 years


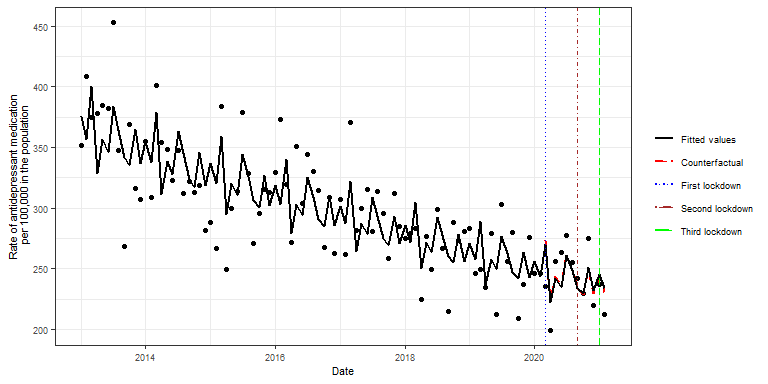


**Supplemental Figure 9.** **Scatterplot and Regression Fitted Values for Adults Aged Over 65 Years**. Scatter plot of the monthly rate of incident antidepressant fills. Black lines: regression fitted values; Red lines: counterfactual. The horizontal lines marked the onset of the first (blue), second (brown) and third lockdown (green) in Israel. Please see Table 2 for statistical values.

# Supplemental Figure 10. Scatterplot and regression fitted values for stl and ma seasonal decompositions


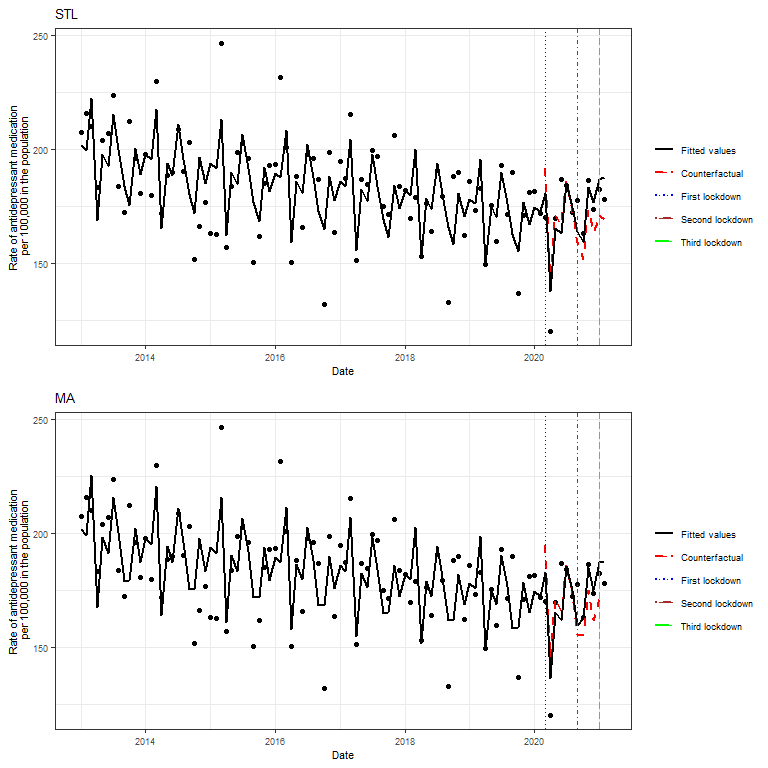


**Supplemental Figure 10. Scatterplot and Regression Fitted Values for STL and MA Seasonal Decompositions**. Top panel: Seasonal and Trend decomposition (STL) using Loess. Lower panel: Moving average (MA) decomposition. Scatter plot of the monthly rate of incident antidepressant fills. Black lines: regression fitted values; Red lines: counterfactual. The horizontal lines marked the onset of the first (blue), second (brown) and third lockdown (green) in Israel. Please see Table 2 for statistical values.

# Supplemental Figure 11. Scatterplot and regression fitted values aggregated in 15 day intervals


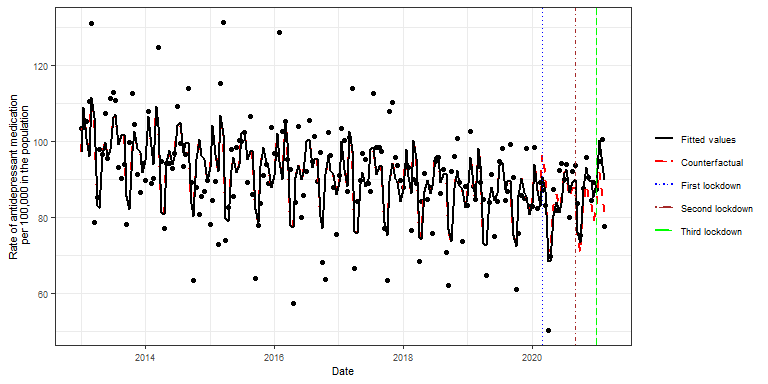


**Supplemental Figure 11. Scatterplot and Regression Fitted Values Aggregated in 15 Day Intervals**. Scatter plot of the monthly rate of incident antidepressant fills. Black lines: regression fitted values; Red lines: counterfactual. The horizontal lines marked the onset of the first (blue), second (brown) and third lockdown (green) in Israel. Please see Table 2 for statistical values.

# Supplemental Figure 12. Scatterplot and regression fitted values for 15 day intervals and lockdown periods


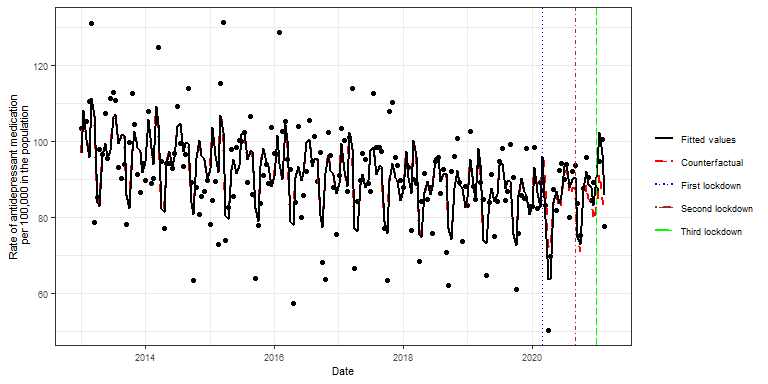


**Supplemental Figure 12. Scatterplot and Regression Fitted Values for 15 Day Intervals and Lockdown Periods.** The scatter plot shows the biweekly rate of antidepressant medications. Black lines: regression fitted values; Red lines: counterfactual. The horizontal lines marked the onset of the first (blue), second (brown) and third lockdown (green) in Israel. Please see Table 2 for statistical values.

# Supplemental Figure 13. Scatterplot and regression fitted values restricted to SARS-CoV-2 non-cases


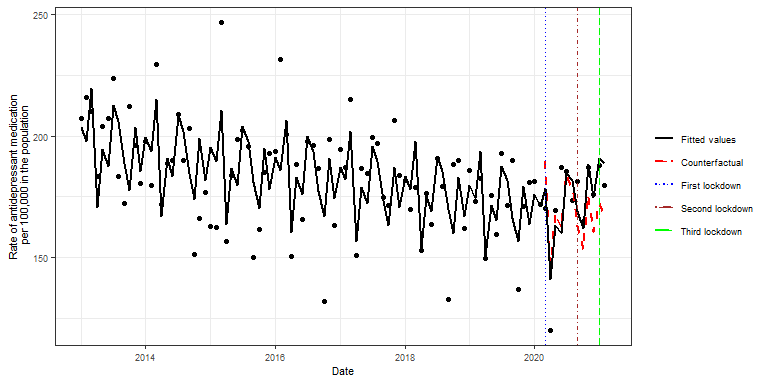


**Supplemental Figure 13. Scatterplot and Regression Fitted Values Restricted to SARS-CoV-2 Non-Cases.** Scatter plot of the monthly rate of incident antidepressant fills. Black lines: regression fitted values; Red lines: counterfactual. The horizontal lines marked the onset of the first (blue), second (brown) and third lockdown (green) in Israel. Please see Table 2 for statistical values.

# Supplemental Figure 14. Scatterplot and regression fitted values for the COVID-19 pandemic and 2014 Gaza war


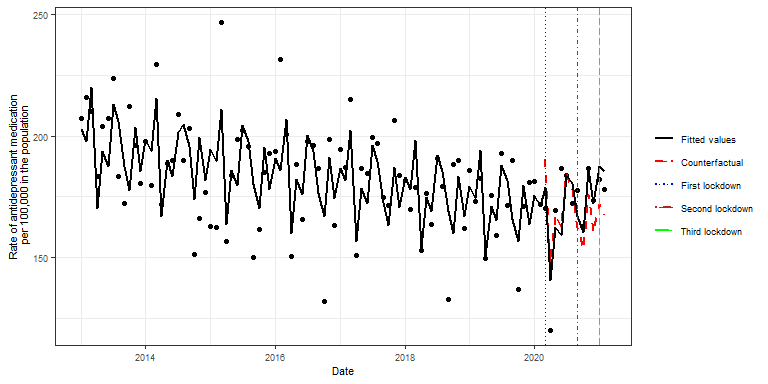


**Supplemental Figure 14. Scatterplot and regression fitted values for the COVID-19 pandemic and 2014 Gaza war.**  Scatter plot of the monthly rate of incident antidepressant fills. Black lines: regression fitted values; Red lines: counterfactual. The horizontal lines marked the onset of the first (blue), second (brown) and third lockdown (green) in Israel. Please see Table 2 for statistical values.

# Supplemental Figure 15. Scatterplot and regression fitted values for the COVID-19 pandemic and 2014 Gaza war by sex


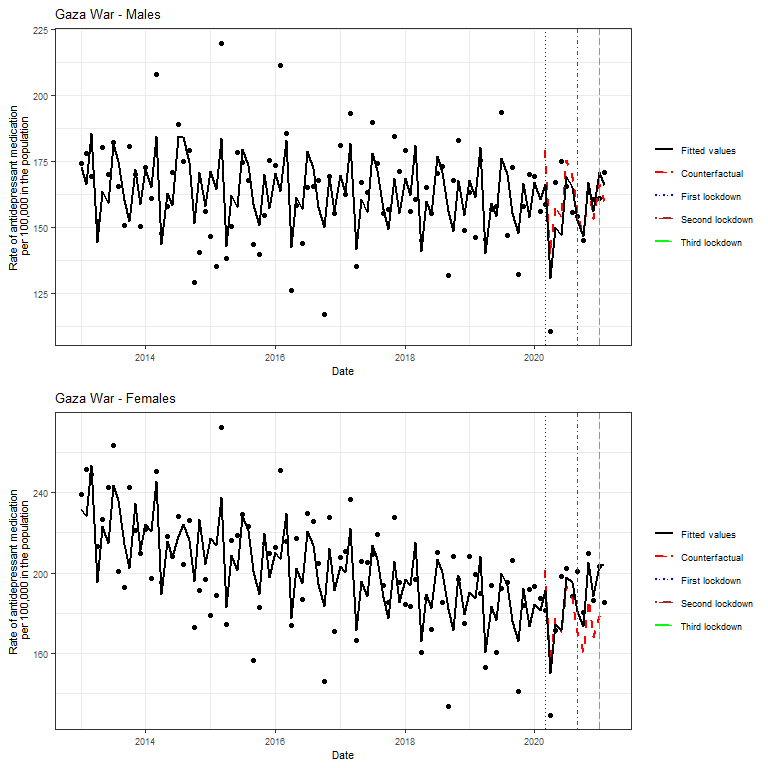


**Supplemental Figure 15. Scatterplot and regression fitted values for the COVID-19 pandemic and 2014 Gaza war by sex.**  Top panel: males; Lower panel: females. Scatter plot of the monthly rate of incident antidepressant fills. Black lines: regression fitted values; Red lines: counterfactual. The horizontal lines marked the onset of the first (blue), second (brown) and third lockdown (green) in Israel. Please see Table 2 for statistical values.

# Supplemental Table 2. Forecasted values and 95% prediction Intervals for the three forecast scenarios

|  | | | |
| --- | --- | --- | --- |
| **Time** | **Scenario 1** | **Scenario 2** | **Scenario 3** |
| March 2021 | 185.76  (183.03, 188.53) | 208.72  (205.66, 211.84) | 176.91  (173.50, 180.39) |
| April 2021 | 144.20  (141.20, 147.27) | 164.44  (161.02, 167.94) | 157.82  (153.51, 162.26) |
| May 2021 | 164.55  (160.34, 168.87) | 190.44  (185.57, 195.44) | 158.82  (153.49, 164.33) |
| June 2021 | 158.52  (153.83, 163.36) | 186.19 (180.68, 191.88) | 168.90  (162.34, 175.71) |
| July 2021 | 179.78  (173.81, 185.95) | 214.30 (207.18, 221.66) | 179.62  (171.81, 187.78) |
| August 2021 | 174.26  (167.90, 180.86) | 210.82  (203.13, 218.80) | 180.75  (172.12, 189.81) |
| September 2021 | 159.25 (152.96, 165.81) | 195.53 (187.80, 203.58) | 161.24  (152.90, 170.04) |
| October 2021 | 150.12  (143.75, 156.76) | 187.05  (179.12, 195.34) | 156.16  (147.50, 165.32) |
| November 2021 | 172.16  (164.39, 180.29) | 217.71  (207.88, 228.00) | 170.59  (160.53, 181.28) |
| December 2021 | 156.87  (149.38, 164.73) | 201.32  (191.72, 211.42) | 167.11  (156.69, 178.22) |
| Note. Three forecasts from 1st March 2021 to 1st December 2021. Scenario: (1) no ongoing effects of the COVID-19 pandemic; (2) ongoing effects of the pandemic; and (3) based on the intervals before and during the pandemic. As a reference, forecasts for December 2021 may be contrasted to past observed values for December 2019 (180.91) and December 2020 (173.54). Multi-step prediction intervals were calculated using the multi-step forecast standard deviation for the drift method. | | | |

# Supplemental Table 3. Complete model summary for the Gaza war

| \| Covariate \| Estimate \| 2.5 % CI \| 97.5 % CI \| t-value \| P-value \| \| --- \| --- \| --- \| --- \| --- \| --- \| \| Level \| -6.2291 \| -6.2674 \| -6.1910 \| -319.59 \| P<0.001 \| \| Slope \| -0.0017 \| -0.0025 \| -0.0010 \| -4.53 \| P<0.001 \| \| Covid-19 Level Change \| -0.0616 \| -0.1633 \| 0.0385 \| -1.20 \| 0.23 \| \| Gaza War Level Change \| -0.0347 \| -0.1955 \| 0.1201 \| -0.43 \| 0.67 \| \| Covid-19 Slope Change \| 0.0149 \| 0.0007 \| 0.0291 \| 2.05 \| 0.04 \| \| Gaza War Slope Change \| 0.0491 \| -0.0724 \| 0.1707 \| 0.79 \| 0.43 \| \| Fourier1 \| 0.0529 \| 0.0283 \| 0.0776 \| 4.22 \| P<0.001 \| \| Fourier2 \| -0.0445 \| -0.0688 \| -0.0202 \| -3.59 \| P<0.001 \| \| Fourier3 \| -0.0306 \| -0.0549 \| -0.0064 \| -2.48 \| 0.02 \| \| Fourier4 \| 0.0299 \| 0.0056 \| 0.0543 \| 2.41 \| 0.02 \| \| Fourier5 \| -0.0406 \| -0.0578 \| -0.0235 \| -4.64 \| P<0.001 \| |
| --- | --- | --- | --- | --- | --- | --- | --- | --- | --- | --- | --- | --- | --- | --- | --- | --- | --- | --- | --- | --- | --- | --- | --- | --- | --- | --- | --- | --- | --- | --- | --- | --- | --- | --- | --- | --- | --- | --- | --- | --- | --- | --- | --- | --- | --- | --- | --- | --- | --- | --- | --- | --- | --- | --- | --- | --- | --- | --- | --- | --- | --- | --- | --- | --- | --- | --- | --- | --- | --- | --- | --- | --- |

# Supplemental References

Bernal, J.L., Cummins, S, & Gasparrini, A. (2017). Interrupted time series regression for the evaluation of public health interventions: a tutorial. *International Journal of Epidemiology*,*46*(1),348-355. doi:[10.1093/ije/dyw098](https://doi.org/10.1093/ije/dyw098)

Hale, T., Angrist, N., Goldszmidt, R., Kira, B., Petherick, A., Phillips, T., …Tatlow, H. (2021). A global panel database of pandemic policies (Oxford COVID-19 government response tracker). *Nature Human Behaviour*, *5*(4),529-538. doi:[10.1038/s41562-021-01079-8](https://doi.org/10.1038/s41562-021-01079-8)

Hyndman, R.J., & Athanasopoulos, G. (2018). *Forecasting: Principles and practice*. (2nd ed.). OTexts.

Levine, S.Z,, Levav, I., Yoffe, R., Becher, Y., & Pugachova, I. (2016). Genocide exposure and subsequent suicide risk: a population-based study. *PLoS ONE*,*11*(2). doi:[10.1371/journal.pone.0149524](https://doi.org/10.1371/journal.pone.0149524)
